# Benzil bis­(ketazine)

**DOI:** 10.1107/S1600536809024489

**Published:** 2009-07-04

**Authors:** Goutam Kumar Patra, Anindita Mukherjee, Seik Weng Ng

**Affiliations:** a Department of Chemistry, Vijaygarh Jyotish Ray College, 8/2 Vijaygarh, Jadavpur, Kolkata 32, India; b Department of Chemistry, University of Malaya, 50603 Kuala Lumpur, Malaysia

## Abstract

The title compound (systematic name: 1,1′,2,2′-tetra­phenyl-2,2′-azinodiethanone), C_28_H_20_N_2_O_2_, was obtained by the reaction of benzil monohydrazone with chromium(III) nitrate. The dibenzyl­idene hydrazine unit is nearly planar (r.m.s. deviation = 0.073 Å) and the two benzoyl units are oriented almost perpendicular to it [dihedral angle = 87.81 (2), 87.81 (2)°]. The mol­ecules are linked into chains along the *c* axis by C—H⋯O hydrogen bonds and the chains are cross-linked *via* C—H⋯π inter­actions involving the benzoyl phenyl rings.

## Related literature

For the synthesis of title compound using copper bis­(acetyl­acetonate) as catalyst, see: Ibata & Singh (1994 ▶); Singh (1983 ▶).
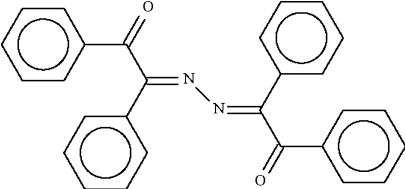


## Experimental

### 

#### Crystal data

C_28_H_20_N_2_O_2_
*M* *_r_* = 416.46
Monoclinic, 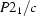

*a* = 8.2875 (2) Å
*b* = 22.1023 (4) Å
*c* = 11.6602 (2) Å
β = 97.539 (1)°
*V* = 2117.37 (7) Å^3^
*Z* = 4
Mo *K*α radiation
μ = 0.08 mm^−1^
*T* = 140 K
0.30 × 0.04 × 0.03 mm

#### Data collection

Bruker SMART APEX area-detector diffractometer
Absorption correction: none
17323 measured reflections
4857 independent reflections
3510 reflections with *I* > 2σ(*I*)
*R*_int_ = 0.030

#### Refinement

*R*[*F* ^2^ > 2σ(*F* ^2^)] = 0.040
*wR*(*F* ^2^) = 0.107
*S* = 1.03
4857 reflections
289 parameters
H-atom parameters constrained
Δρ_max_ = 0.24 e Å^−3^
Δρ_min_ = −0.21 e Å^−3^

### 

Data collection: *APEX2* (Bruker, 2008 ▶); cell refinement: *SAINT* (Bruker, 2008 ▶); data reduction: *SAINT*; program(s) used to solve structure: *SHELXS97* (Sheldrick, 2008 ▶); program(s) used to refine structure: *SHELXL97* (Sheldrick, 2008 ▶); molecular graphics: *X-SEED* (Barbour, 2001 ▶); software used to prepare material for publication: *publCIF* (Westrip, 2009 ▶).

## Supplementary Material

Crystal structure: contains datablocks global, I. DOI: 10.1107/S1600536809024489/ci2835sup1.cif

Structure factors: contains datablocks I. DOI: 10.1107/S1600536809024489/ci2835Isup2.hkl

Additional supplementary materials:  crystallographic information; 3D view; checkCIF report

## Figures and Tables

**Table 1:** Hydrogen-bond geometry (Å, °)

*D*—H⋯*A*	*D*—H	H⋯*A*	*D*⋯*A*	*D*—H⋯*A*
C1—H1⋯O1^i^	0.95	2.24	3.0832 (16)	147
C13—H13⋯*Cg*1^ii^	0.95	2.68	3.5705 (16)	157
C18—H18⋯*Cg*2^iii^	0.95	2.66	3.5304 (16)	153

Symmetry codes: (i) 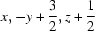; (ii) 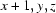; (iii) 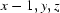. *Cg*1 and *Cg*2 are centroids of the C1–C6 and C23–C28 rings, respectively.

**Fig. 1.**
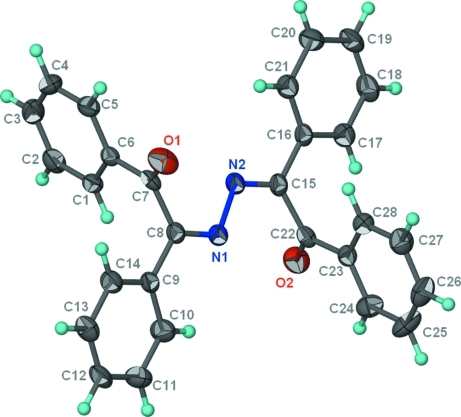
Displacement ellipsoid plot (Barbour, 2001) of C28H20N2O2 at the 70% probability level; H atoms are drawn as spheres of arbitrary radius.

## supplementary crystallographic information

### Experimental

Benzil monohydrazone (0.224 g, 1 mmol) was dissolved in acetonitrile (20 ml) and to this was added chromium nitrate nonahyrate (0.40 g, 1 mmol). The greenish yellowish mixture was stirred at room temperature for 6 h. Bright yellow prisms were collected in 40% yield.

### Refinement

H-atoms were placed in calculated positions (C-H = 0.95 Å) and were included in the refinement in the riding model approximation, with *U_iso_*(H) set to 1.2*U_eq_*(C).

### Crystal data

**Table:**

C_28_H_20_N_2_O_2_	*F*(000) = 872
*M**_r_* = 416.46	*D*_x_ = 1.306 Mg m^−^^3^
Monoclinic, *P*2_1_/*c*	Mo *K*α radiation, λ = 0.71073 Å
Hall symbol: -P 2ybc	Cell parameters from 3878 reflections
*a* = 8.2875 (2) Å	θ = 2.5–27.9°
*b* = 22.1023 (4) Å	µ = 0.08 mm^−^^1^
*c* = 11.6602 (2) Å	*T* = 140 K
β = 97.539 (1)°	Prism, yellow
*V* = 2117.37 (7) Å^3^	0.30 × 0.04 × 0.03 mm
*Z* = 4	

### Data collection

**Table:**

Bruker SMART APEX area-detector diffractometer	3510 reflections with *I* > 2σ(*I*)
Radiation source: fine-focus sealed tube	*R*_int_ = 0.030
graphite	θ_max_ = 27.5°, θ_min_ = 1.8°
ω scans	*h* = −10→10
17323 measured reflections	*k* = −28→27
4857 independent reflections	*l* = −15→15

### Refinement

**Table:**

Refinement on *F*^2^	Primary atom site location: structure-invariant direct methods
Least-squares matrix: full	Secondary atom site location: difference Fourier map
*R*[*F*^2^ > 2σ(*F*^2^)] = 0.040	Hydrogen site location: inferred from neighbouring sites
*wR*(*F*^2^) = 0.107	H-atom parameters constrained
*S* = 1.02	*w* = 1/[σ^2^(*F*_o_^2^) + (0.0486*P*)^2^ + 0.4715*P*] where *P* = (*F*_o_^2^ + 2*F*_c_^2^)/3
4857 reflections	(Δ/σ)_max_ = 0.001
289 parameters	Δρ_max_ = 0.24 e Å^−^^3^
0 restraints	Δρ_min_ = −0.21 e Å^−^^3^

### Fractional atomic coordinates and isotropic or equivalent isotropic displacement parameters (Å^2^)

**Table:**

	*x*	*y*	*z*	*U*_iso_*/*U*_eq_	
O1	0.37058 (13)	0.75630 (5)	0.13158 (8)	0.0366 (3)	
O2	0.17265 (13)	0.56277 (5)	0.47749 (8)	0.0336 (3)	
N1	0.34699 (13)	0.66169 (5)	0.33697 (11)	0.0257 (3)	
N2	0.18382 (13)	0.66513 (5)	0.28286 (10)	0.0252 (3)	
C1	0.27649 (16)	0.81384 (6)	0.40407 (11)	0.0215 (3)	
H1	0.3294	0.7835	0.4531	0.026*	
C2	0.20231 (17)	0.86230 (7)	0.45113 (13)	0.0303 (3)	
H2	0.2043	0.8653	0.5326	0.036*	
C3	0.12519 (19)	0.90640 (7)	0.37938 (15)	0.0377 (4)	
H3	0.0744	0.9397	0.4118	0.045*	
C4	0.12170 (19)	0.90223 (7)	0.26063 (15)	0.0370 (4)	
H4	0.0678	0.9325	0.2119	0.044*	
C5	0.19592 (17)	0.85447 (6)	0.21267 (13)	0.0278 (3)	
H5	0.1945	0.8520	0.1312	0.033*	
C6	0.27335 (15)	0.80970 (6)	0.28467 (11)	0.0198 (3)	
C7	0.35415 (15)	0.75841 (6)	0.23347 (11)	0.0219 (3)	
C8	0.42829 (16)	0.70877 (6)	0.31371 (11)	0.0211 (3)	
C9	0.59933 (16)	0.71554 (6)	0.36520 (11)	0.0211 (3)	
C10	0.66251 (18)	0.67959 (7)	0.45829 (13)	0.0318 (3)	
H10	0.5949	0.6504	0.4881	0.038*	
C11	0.82226 (18)	0.68586 (7)	0.50792 (14)	0.0349 (4)	
H11	0.8639	0.6612	0.5717	0.042*	
C12	0.92181 (17)	0.72815 (7)	0.46459 (13)	0.0296 (3)	
H12	1.0319	0.7324	0.4985	0.036*	
C13	0.86090 (17)	0.76404 (7)	0.37219 (13)	0.0312 (3)	
H13	0.9294	0.7929	0.3422	0.037*	
C14	0.70016 (17)	0.75823 (6)	0.32287 (12)	0.0267 (3)	
H14	0.6586	0.7835	0.2599	0.032*	
C15	0.10218 (16)	0.61745 (6)	0.30204 (11)	0.0215 (3)	
C16	−0.06870 (16)	0.61175 (6)	0.24937 (11)	0.0215 (3)	
C17	−0.16874 (17)	0.56676 (6)	0.28567 (13)	0.0279 (3)	
H17	−0.1274	0.5398	0.3460	0.033*	
C18	−0.32831 (18)	0.56134 (7)	0.23374 (14)	0.0327 (3)	
H18	−0.3961	0.5306	0.2586	0.039*	
C19	−0.38917 (17)	0.60026 (7)	0.14627 (13)	0.0307 (3)	
H19	−0.4987	0.5962	0.1110	0.037*	
C20	−0.29147 (17)	0.64511 (7)	0.10965 (13)	0.0302 (3)	
H20	−0.3338	0.6720	0.0495	0.036*	
C21	−0.13179 (17)	0.65073 (6)	0.16090 (12)	0.0269 (3)	
H21	−0.0646	0.6815	0.1355	0.032*	
C22	0.17830 (16)	0.56479 (6)	0.37387 (11)	0.0222 (3)	
C23	0.25313 (16)	0.51611 (6)	0.31005 (12)	0.0215 (3)	
C24	0.34362 (17)	0.47038 (7)	0.37097 (13)	0.0299 (3)	
H24	0.3554	0.4697	0.4531	0.036*	
C25	0.4161 (2)	0.42613 (7)	0.31128 (15)	0.0372 (4)	
H25	0.4800	0.3956	0.3528	0.045*	
C26	0.39637 (19)	0.42599 (7)	0.19180 (14)	0.0344 (4)	
H26	0.4459	0.3952	0.1516	0.041*	
C27	0.30485 (18)	0.47042 (7)	0.13063 (13)	0.0309 (3)	
H27	0.2903	0.4700	0.0484	0.037*	
C28	0.23428 (17)	0.51564 (6)	0.18982 (12)	0.0242 (3)	
H28	0.1725	0.5466	0.1478	0.029*	

### Atomic displacement parameters (Å^2^)

**Table:**

	*U*^11^	*U*^22^	*U*^33^	*U*^12^	*U*^13^	*U*^23^
O1	0.0392 (6)	0.0513 (7)	0.0191 (5)	0.0020 (5)	0.0023 (4)	−0.0069 (5)
O2	0.0389 (6)	0.0379 (6)	0.0238 (5)	0.0007 (5)	0.0037 (4)	−0.0048 (4)
N1	0.0177 (6)	0.0207 (6)	0.0374 (7)	−0.0007 (4)	−0.0007 (5)	−0.0034 (5)
N2	0.0167 (6)	0.0214 (6)	0.0366 (7)	−0.0006 (4)	0.0000 (5)	−0.0056 (5)
C1	0.0188 (6)	0.0230 (6)	0.0220 (7)	−0.0012 (5)	−0.0003 (5)	0.0008 (5)
C2	0.0244 (7)	0.0341 (8)	0.0317 (8)	−0.0013 (6)	0.0009 (6)	−0.0103 (6)
C3	0.0286 (8)	0.0273 (8)	0.0549 (11)	0.0056 (6)	−0.0034 (7)	−0.0120 (7)
C4	0.0334 (8)	0.0229 (7)	0.0501 (10)	0.0023 (6)	−0.0119 (7)	0.0057 (7)
C5	0.0265 (7)	0.0266 (7)	0.0278 (7)	−0.0057 (6)	−0.0051 (6)	0.0066 (6)
C6	0.0162 (6)	0.0200 (6)	0.0227 (7)	−0.0044 (5)	0.0004 (5)	0.0012 (5)
C7	0.0171 (6)	0.0267 (7)	0.0206 (6)	−0.0055 (5)	−0.0017 (5)	−0.0039 (5)
C8	0.0199 (7)	0.0202 (6)	0.0230 (7)	−0.0002 (5)	0.0026 (5)	−0.0061 (5)
C9	0.0187 (6)	0.0207 (6)	0.0238 (7)	−0.0006 (5)	0.0018 (5)	−0.0055 (5)
C10	0.0245 (7)	0.0319 (8)	0.0380 (8)	−0.0049 (6)	−0.0002 (6)	0.0069 (6)
C11	0.0291 (8)	0.0367 (8)	0.0363 (8)	0.0007 (6)	−0.0058 (7)	0.0049 (7)
C12	0.0178 (7)	0.0347 (8)	0.0350 (8)	−0.0014 (6)	−0.0014 (6)	−0.0115 (6)
C13	0.0231 (7)	0.0346 (8)	0.0358 (8)	−0.0082 (6)	0.0029 (6)	−0.0029 (6)
C14	0.0244 (7)	0.0276 (7)	0.0275 (7)	−0.0033 (6)	0.0010 (6)	−0.0008 (6)
C15	0.0206 (7)	0.0192 (6)	0.0252 (7)	−0.0008 (5)	0.0045 (5)	−0.0066 (5)
C16	0.0197 (6)	0.0192 (6)	0.0261 (7)	−0.0010 (5)	0.0045 (5)	−0.0064 (5)
C17	0.0244 (7)	0.0256 (7)	0.0332 (8)	−0.0025 (6)	0.0025 (6)	−0.0012 (6)
C18	0.0242 (7)	0.0331 (8)	0.0413 (9)	−0.0104 (6)	0.0064 (6)	−0.0038 (7)
C19	0.0185 (7)	0.0386 (8)	0.0343 (8)	−0.0021 (6)	0.0002 (6)	−0.0102 (6)
C20	0.0254 (7)	0.0341 (8)	0.0300 (8)	0.0014 (6)	−0.0004 (6)	−0.0013 (6)
C21	0.0232 (7)	0.0257 (7)	0.0320 (8)	−0.0030 (6)	0.0041 (6)	−0.0009 (6)
C22	0.0180 (6)	0.0241 (7)	0.0240 (7)	−0.0051 (5)	0.0013 (5)	−0.0032 (5)
C23	0.0186 (6)	0.0189 (6)	0.0270 (7)	−0.0030 (5)	0.0027 (5)	−0.0001 (5)
C24	0.0288 (8)	0.0319 (8)	0.0295 (8)	0.0032 (6)	0.0060 (6)	0.0076 (6)
C25	0.0386 (9)	0.0268 (8)	0.0485 (10)	0.0117 (7)	0.0140 (8)	0.0129 (7)
C26	0.0374 (9)	0.0232 (7)	0.0455 (9)	0.0040 (6)	0.0166 (7)	−0.0017 (6)
C27	0.0326 (8)	0.0313 (8)	0.0295 (8)	0.0000 (6)	0.0063 (6)	−0.0050 (6)
C28	0.0238 (7)	0.0221 (7)	0.0264 (7)	0.0009 (5)	0.0016 (6)	0.0000 (5)

### Geometric parameters (Å, °)

**Table:**

O1—C7	1.2143 (16)	C13—H13	0.95
O2—C22	1.2160 (16)	C14—H14	0.95
N1—C8	1.2878 (17)	C15—C16	1.4733 (18)
N1—N2	1.4173 (15)	C15—C22	1.5216 (19)
N2—C15	1.2877 (17)	C16—C21	1.3922 (19)
C1—C2	1.3839 (19)	C16—C17	1.3955 (19)
C1—C6	1.3920 (18)	C17—C18	1.385 (2)
C1—H1	0.95	C17—H17	0.95
C2—C3	1.385 (2)	C18—C19	1.378 (2)
C2—H2	0.95	C18—H18	0.95
C3—C4	1.384 (2)	C19—C20	1.383 (2)
C3—H3	0.95	C19—H19	0.95
C4—C5	1.377 (2)	C20—C21	1.384 (2)
C4—H4	0.95	C20—H20	0.95
C5—C6	1.3980 (18)	C21—H21	0.95
C5—H5	0.95	C22—C23	1.4890 (18)
C6—C7	1.4814 (18)	C23—C28	1.3902 (19)
C7—C8	1.5185 (18)	C23—C24	1.3959 (19)
C8—C9	1.4727 (18)	C24—C25	1.383 (2)
C9—C10	1.3908 (19)	C24—H24	0.95
C9—C14	1.3929 (19)	C25—C26	1.381 (2)
C10—C11	1.380 (2)	C25—H25	0.95
C10—H10	0.95	C26—C27	1.381 (2)
C11—C12	1.385 (2)	C26—H26	0.95
C11—H11	0.95	C27—C28	1.3869 (19)
C12—C13	1.379 (2)	C27—H27	0.95
C12—H12	0.95	C28—H28	0.95
C13—C14	1.386 (2)		
			
C8—N1—N2	110.97 (11)	C9—C14—H14	119.9
C15—N2—N1	111.78 (11)	N2—C15—C16	119.72 (12)
C2—C1—C6	119.77 (13)	N2—C15—C22	122.16 (12)
C2—C1—H1	120.1	C16—C15—C22	118.06 (11)
C6—C1—H1	120.1	C21—C16—C17	119.04 (12)
C1—C2—C3	119.93 (14)	C21—C16—C15	120.20 (12)
C1—C2—H2	120.0	C17—C16—C15	120.75 (12)
C3—C2—H2	120.0	C18—C17—C16	120.00 (14)
C4—C3—C2	120.32 (14)	C18—C17—H17	120.0
C4—C3—H3	119.8	C16—C17—H17	120.0
C2—C3—H3	119.8	C19—C18—C17	120.34 (13)
C5—C4—C3	120.37 (14)	C19—C18—H18	119.8
C5—C4—H4	119.8	C17—C18—H18	119.8
C3—C4—H4	119.8	C18—C19—C20	120.25 (13)
C4—C5—C6	119.54 (14)	C18—C19—H19	119.9
C4—C5—H5	120.2	C20—C19—H19	119.9
C6—C5—H5	120.2	C19—C20—C21	119.76 (14)
C1—C6—C5	120.06 (13)	C19—C20—H20	120.1
C1—C6—C7	120.21 (12)	C21—C20—H20	120.1
C5—C6—C7	119.71 (12)	C20—C21—C16	120.61 (13)
O1—C7—C6	122.57 (13)	C20—C21—H21	119.7
O1—C7—C8	119.13 (12)	C16—C21—H21	119.7
C6—C7—C8	118.18 (11)	O2—C22—C23	122.88 (13)
N1—C8—C9	119.89 (12)	O2—C22—C15	120.51 (12)
N1—C8—C7	122.21 (12)	C23—C22—C15	116.58 (11)
C9—C8—C7	117.89 (11)	C28—C23—C24	119.31 (12)
C10—C9—C14	118.78 (12)	C28—C23—C22	120.68 (12)
C10—C9—C8	120.25 (12)	C24—C23—C22	120.01 (12)
C14—C9—C8	120.96 (12)	C25—C24—C23	119.74 (14)
C11—C10—C9	120.80 (14)	C25—C24—H24	120.1
C11—C10—H10	119.6	C23—C24—H24	120.1
C9—C10—H10	119.6	C26—C25—C24	120.49 (14)
C10—C11—C12	119.94 (14)	C26—C25—H25	119.8
C10—C11—H11	120.0	C24—C25—H25	119.8
C12—C11—H11	120.0	C25—C26—C27	120.26 (14)
C13—C12—C11	119.90 (13)	C25—C26—H26	119.9
C13—C12—H12	120.0	C27—C26—H26	119.9
C11—C12—H12	120.0	C26—C27—C28	119.63 (14)
C12—C13—C14	120.27 (14)	C26—C27—H27	120.2
C12—C13—H13	119.9	C28—C27—H27	120.2
C14—C13—H13	119.9	C23—C28—C27	120.55 (13)
C13—C14—C9	120.30 (13)	C23—C28—H28	119.7
C13—C14—H14	119.9	C27—C28—H28	119.7
			
C8—N1—N2—C15	177.82 (12)	N1—N2—C15—C16	−177.87 (11)
C6—C1—C2—C3	0.1 (2)	N1—N2—C15—C22	−0.55 (17)
C1—C2—C3—C4	0.1 (2)	N2—C15—C16—C21	13.52 (19)
C2—C3—C4—C5	−0.5 (2)	C22—C15—C16—C21	−163.92 (12)
C3—C4—C5—C6	0.8 (2)	N2—C15—C16—C17	−167.62 (13)
C2—C1—C6—C5	0.20 (19)	C22—C15—C16—C17	14.95 (18)
C2—C1—C6—C7	179.23 (12)	C21—C16—C17—C18	0.1 (2)
C4—C5—C6—C1	−0.6 (2)	C15—C16—C17—C18	−178.82 (13)
C4—C5—C6—C7	−179.66 (12)	C16—C17—C18—C19	−0.1 (2)
C1—C6—C7—O1	−171.53 (13)	C17—C18—C19—C20	−0.1 (2)
C5—C6—C7—O1	7.51 (19)	C18—C19—C20—C21	0.2 (2)
C1—C6—C7—C8	4.51 (18)	C19—C20—C21—C16	−0.2 (2)
C5—C6—C7—C8	−176.45 (12)	C17—C16—C21—C20	0.1 (2)
N2—N1—C8—C9	177.59 (11)	C15—C16—C21—C20	178.98 (12)
N2—N1—C8—C7	−2.72 (17)	N2—C15—C22—O2	91.73 (16)
O1—C7—C8—N1	−93.45 (16)	C16—C15—C22—O2	−90.91 (15)
C6—C7—C8—N1	90.38 (15)	N2—C15—C22—C23	−90.28 (15)
O1—C7—C8—C9	86.25 (15)	C16—C15—C22—C23	87.09 (14)
C6—C7—C8—C9	−89.92 (14)	O2—C22—C23—C28	169.79 (13)
N1—C8—C9—C10	−15.03 (19)	C15—C22—C23—C28	−8.15 (18)
C7—C8—C9—C10	165.26 (12)	O2—C22—C23—C24	−10.4 (2)
N1—C8—C9—C14	165.69 (13)	C15—C22—C23—C24	171.70 (12)
C7—C8—C9—C14	−14.01 (18)	C28—C23—C24—C25	1.4 (2)
C14—C9—C10—C11	−0.2 (2)	C22—C23—C24—C25	−178.43 (13)
C8—C9—C10—C11	−179.45 (13)	C23—C24—C25—C26	−1.6 (2)
C9—C10—C11—C12	−0.3 (2)	C24—C25—C26—C27	0.5 (2)
C10—C11—C12—C13	0.2 (2)	C25—C26—C27—C28	0.7 (2)
C11—C12—C13—C14	0.4 (2)	C24—C23—C28—C27	−0.2 (2)
C12—C13—C14—C9	−0.8 (2)	C22—C23—C28—C27	179.65 (13)
C10—C9—C14—C13	0.7 (2)	C26—C27—C28—C23	−0.9 (2)
C8—C9—C14—C13	−179.97 (13)		

### Hydrogen-bond geometry (Å, °)

**Table:**

*D*—H···*A*	*D*—H	H···*A*	*D*···*A*	*D*—H···*A*
C1—H1···O1^i^	0.95	2.24	3.0832 (16)	147
C13—H13···Cg1^ii^	0.95	2.68	3.5705 (16)	157
C18—H18···Cg2^iii^	0.95	2.66	3.5304 (16)	153

Symmetry codes: (i) *x*, −*y*+3/2, *z*+1/2; (ii) *x*+1, *y*, *z*; (iii) *x*−1, *y*, *z*.
